# Bridging Microbial Functional Traits With Localized Process Rates at Soil Interfaces

**DOI:** 10.3389/fmicb.2021.625697

**Published:** 2021-10-28

**Authors:** Evgenia Blagodatskaya, Mika Tarkka, Claudia Knief, Robert Koller, Stephan Peth, Volker Schmidt, Sandra Spielvogel, Daniel Uteau, Matthias Weber, Bahar S. Razavi

**Affiliations:** ^1^Department of Soil Ecology, Helmholtz Centre for Environmental Research, Halle (Saale), Germany; ^2^Agro-Technological Institute, RUDN University, Moscow, Russia; ^3^German Centre for Integrative Biodiversity Research Halle–Jena–Leipzig, Leipzig, Germany; ^4^Institute of Crop Science and Resource Conservation – Molecular Biology of the Rhizosphere, University of Bonn, Bonn, Germany; ^5^Institute of Bio- and Geosciences, IBG-2: Plant Sciences, Forschungszentrum Jülich GmbH, Jülich, Germany; ^6^Institute of Soil Science, University of Hannover, Hanover, Germany; ^7^Institute of Stochastics, Ulm University, Ulm, Germany; ^8^Department Soil Science, Institute for Plant Nutrition and Soil Science, Christian-Albrechts University Kiel, Kiel, Germany; ^9^Department of Soil Science, Faculty of Organic Agricultural Sciences, University of Kassel, Kassel, Germany; ^10^Department of Soil and Plant Microbiome, Institute of Phytopathology, Christian-Albrechts-University of Kiel, Kiel, Germany

**Keywords:** rhizosphere, mycorrhizosphere, detritusphere, (bio)-pores, soil aggregates, soil priming, trophic interactions, statistical analysis of process locations

## Abstract

In this review, we introduce microbially-mediated soil processes, players, their functional traits, and their links to processes at biogeochemical interfaces [e.g., rhizosphere, detritusphere, (bio)-pores, and aggregate surfaces]. A conceptual view emphasizes the central role of the rhizosphere in interactions with other biogeochemical interfaces, considering biotic and abiotic dynamic drivers. We discuss the applicability of three groups of traits based on microbial physiology, activity state, and genomic functional traits to reflect microbial growth in soil. The sensitivity and credibility of modern molecular approaches to estimate microbial-specific growth rates require further development. A link between functional traits determined by physiological (e.g., respiration, biomarkers) and genomic (e.g., genome size, number of ribosomal gene copies per genome, expression of catabolic versus biosynthetic genes) approaches is strongly affected by environmental conditions such as carbon, nutrient availability, and ecosystem type. Therefore, we address the role of soil physico-chemical conditions and trophic interactions as drivers of microbially-mediated soil processes at relevant scales for process localization. The strengths and weaknesses of current approaches (destructive, non-destructive, and predictive) for assessing process localization and the corresponding estimates of process rates are linked to the challenges for modeling microbially-mediated processes in heterogeneous soil microhabitats. Finally, we introduce a conceptual self-regulatory mechanism based on the flexible structure of active microbial communities. Microbial taxa best suited to each successional stage of substrate decomposition become dominant and alter the community structure. The rates of decomposition of organic compounds, therefore, are dependent on the functional traits of dominant taxa and microbial strategies, which are selected and driven by the local environment.

## Relevant Microbially-Mediated Soil Processes

In terrestrial ecosystems, the most critical biochemical processes are performed by soil microorganisms (Brussaard, 2012; Fierer, 2017), and a broad range of microbial functions contribute to essential ecosystem services, such as soil fertility, resilience, and resistance to abiotic and biotic stresses (Mulder et al., 2011). One major category of microbial functions in terrestrial ecosystems is the decomposition and transformation of organic compounds entering the soil, predominantly as plant materials. The majority of microorganisms are capable of breaking down labile compounds derived from fresh plant litter or rhizodeposits, thus ensuring functional redundancy. Other processes rely on more specialized microorganisms in the breakdown of recalcitrant compounds, which usually occur at later stages of organic matter decomposition (Bastian et al., 2009; Pepe-Ranney et al., 2016). Functional redundancy in the soil microbiome, or more generally biodiversity, provides ecosystem resilience (Fierer, 2017; Louca et al., 2018; Maron et al., 2018) and is crucial for ecosystem multifunctionality (Wagg et al., 2014, 2019).

The *primary* input organic substances in soil are microbially transformed into cell constituents or excreted by cells as labile metabolic products (Bradford, 2016). Moreover, microbial respiration during the transformation of organic material results in carbon loss from the soil and atmospheric CO_2_ emissions. An essential fraction of organic C that is assimilated within microbial biomass is further re-utilized after microbial death by multi-stage microbial succession (Morriën, 2016). Plant-derived resources are then transferred to the microbial food web (Kramer et al., 2016; Hünninghaus et al., 2019). Products from living microorganisms, particularly residues of dead microorganisms (necromass), also serve as a *secondary* source of soil organic substrates, finally resulting in sequestration of up to 40% of primary C input (Miltner et al., 2012; Kallenbach et al., 2016; Buckeridge et al., 2020).

Microorganisms decompose organic substrates to maintain their metabolic requirements and enable their growth. Microbial growth and anabolic reactions require not only C and energy, but also a general stoichiometric composition of nutrients (e.g., N and P), which microorganisms have to mobilize from a multiphase (gaseous, liquid, and solid) soil environment (Zechmeister-Boltenstern et al., 2015). If their own stoichiometric requirements are fulfilled, they can release nutrients, thereby increasing the availability to plants (Hodge et al., 2000; Griffiths et al., 2012). Thus, the metabolic activity of soil microorganisms can cause both positive and negative consequences at the ecosystem level, such as (i) C sequestration and losses during decomposition and transformation of soil organic matter (SOM) or (2) nutrient mobilization, possibly followed by losses through leaching of mineral nitrogen and phosphates. These processes can also cause greenhouse gas (N_2_O, CO_2_, and CH_4_) emissions. The direction and intensity of the consequences of microbial metabolic activity are dependent on the functional traits of the organisms performing the ecologically-relevant processes below-ground and the abiotic and biotic conditions these organisms encounter in their habitats.

## Relevant Players, Functional Traits, and Links to Processes

Even though the *active* fraction of a predominantly *dormant* microbial community can be small in nutrient-poor or stressed environments (Jones and Lennon, 2010; Barnard et al., 2013), soil microorganisms are among the most abundant players in the process of decomposition and transformation of SOM (McGuire and Treseder, 2010). Fresh input of labile organic substrates, e.g., in the rhizosphere by rhizodeposition, may enormously increase the fraction of active microorganisms (Blagodatsky et al., 2000) and therewith the decomposition of SOM, thus causing the well-known “rhizosphere priming effect” (Arsjad and Giddens, 1966; Cheng et al., 2003). Moreover, both SOM stabilization and destabilization in the rhizosphere are driven by different processes, not only rhizodeposition, but also root turnover, as well as nutrient and water uptake by plants (Dijkstra et al., 2020).

### Metrics Used to Distinguish Microbial Traits

**FIGURE 1:**
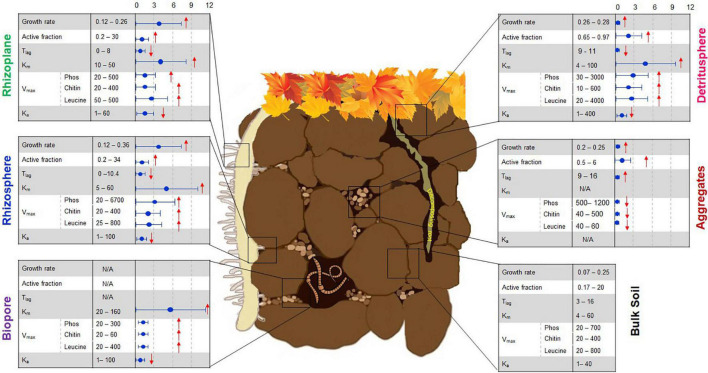
Linking microbial functional traits to process rates in the soil microbial hotspots. First column **(left)**: Growth rate (h^–1^), active fraction (% of total biomass), lag-time, T_lag_ (h), Functional genes (log copies/g dry soil), K_m_, enzyme affinity to the substrate (μmol g^–1^ soil), V_max_, enzyme activity (Phos, phosphatase; Chitin, chitinase; Leucine, leucine-aminopeptidase; nmol g^–1^ h^–1^), K_a_ (h^–1^). Column **(middle)**: ranges of original values based on literature, the column **(right)** shows standardized times of changes in comparison with bulk soil “0, 3, 6, 9, 12.” The arrows show the increased ↑ or decreased ↓ trend compared to bulk soil. References used for this figure have been published as Supplementary Data Sheet 1. Please note that only experiments and analyses performed in soil as matrix were included. After modification from © Nature Education 2012.

Soil organic matter transformation processes mainly rely on the production of extracellular enzymes that facilitate the oxidation or hydrolysis of diverse and complex SOM compounds (Nannipieri et al., 2012). The decomposition rate of SOM is mediated by the molecular nature of SOM as well as by the degree of biotic interactions (Sokol et al., 2018). Moreover, it depends on microbial community traits (DeAngelis et al., 2009), which can be subdivided into three groups (Figure 1). Microbial traits in the first group are very dynamic, for example, the size of the microbial fraction maintaining activity or alert state (*active* biomass) and the time required for dormant microorganisms to switch to active growth (i.e., *lag time*). The second group represents *intrinsic functional traits* of the microbial population, such as maximal specific growth rate (μ*_m_*), generation time (*T*_g_), and affinity of extracellular enzyme systems (*K*_m_) to soil organic substrates used for microbial growth. The third group refers to phenotypic traits at the level of functional genes, for example, those related to internal microbial metabolism, extracellular resource acquisition, or stress tolerance.

### Microbial Traits and Life Strategy Concepts

Bimodal classical concepts based on functional traits (Panikov, 1995; Morris and Blackwood, 2007) exploit the general ecological principles that classify microorganisms, for example, resource requirements (copiotrophs versus oligotrophs), spatial mobility (zymogenous vs. autochtonous), or by growth and efficiency (r- versus K-strategists). These concepts are often not capable of covering a great diversity of microbial functions and life strategies. They have thus been complemented by other concepts such as the competitor-ruderal-stress-tolerator life strategy concept (Krause et al., 2014; Ho et al., 2017), which has very recently been further developed into a “high yield, resource acquisition, and stress tolerance” concept (Malik et al., 2020). However, it remains challenging to identify proxies for specific traits that can serve as quantitative measures of a category. For example, r- and K- strategists can be differentiated by the values of maximum specific growth rates (μ*_m_*), and by enzyme affinities for a substrate (*K*_m_), experimentally determined under *in situ* soil conditions (Blagodatskaya et al., 2014; Tian et al., 2020). In contrast, quantitative estimation of traits specifically responsible for stress tolerance or resource acquisition remains challenging.

### Linking Gene Phenotypic Traits and Microbial Physiology to Estimate Growth Rates

Recent developments in molecular approaches have provided potential for microbial trait differentiation based on information regarding genome size, number of ribosomal gene copies per genome, and quantification of functional marker genes or their transcripts by -omics approaches (Li et al., 2019; Malik et al., 2020). The idea that multiple versus single *rrn* operons in a genome corresponds to faster versus slower growing taxa raises the question of how to relate gene copy number (single vs. multi-copy genes) encoding certain functions with microbial ability to grow under *in situ* soil conditions. At the physiological level, growth rate is dependent on a balance between two fractions of the total proteome (Scott et al., 2014), which are growth-independent (i.e., related to maintenance function) and growth-dependent (i.e., ribosomal and metabolic proteins). In accordance with this concept, a physiological approach based on substrate-induced growth respiration (SIGR) estimates microbial-specific growth rates on glucose, considering the partitioning of microbial respiration into growth-related and growth-independent fractions (Panikov, 1995). As the SIGR approach estimates non-limited microbial growth in soil enabling access to glucose and nutrients, a complementary approach has been developed to determine bacterial and fungal growth by incorporating trace amounts of labeled ^3^H-leucine, ^3^H-thymidine, or ^14^C-acetate (Bååth, 2001; Bååth et al., 2001). Thus, combining molecular and respiratory approaches with isotopic labeling is promising for linking genetic and metabolic potential with microbial functions. However, synchronization of experimental design by sampling time is required for correct comparisons of microbial growth rates obtained by different approaches (see the section “Relevant Approaches for Process Localization”).

A larger genome size corresponds to slower growth induced by glucose; this relationship is weakened in both non-amended and nutrient-rich soil, thus indicating a regulatory role of the environment under natural soil conditions (Li et al., 2019). Reduced growth rates under either C or nutrient limitation may cause contrasting responses of genes involved in bacterial metabolism. Thus, the expression of C catabolic genes increases with decreasing growth rates under C limitation, but decreases under N limitation (You et al., 2013). In contrast, the expression of biosynthetic genes exhibit the opposite growth-rate dependence as catabolic genes. Under environmental control, the growth rates of individual taxa can vary by a factor of two in non-supplemented soils of contrasting ecosystems (Morrissey et al., 2018).

Therefore, environmental selection results in the activation of populations with *intrinsic functional traits* that are mostly suited to the individual microhabitat within heterogeneous soil pore spaces. Thus, beyond the quality and regularity of substrate input, biotic and abiotic environments (Huang et al., 2014), such as soil structure (Berg and Smalla, 2009), presence of organisms (Scheffknecht et al., 2006), and nutritional status (Hinsinger, 2001; Jones et al., 2004) affect microbial functional traits in contrasting soil habitats. In soils covered by vegetation, microbial functional traits are also affected by the physiological and morphological *traits of plant*s.

## Rhizosphere Processes and Interactions Within and Between Interfaces

**FIGURE 2:**
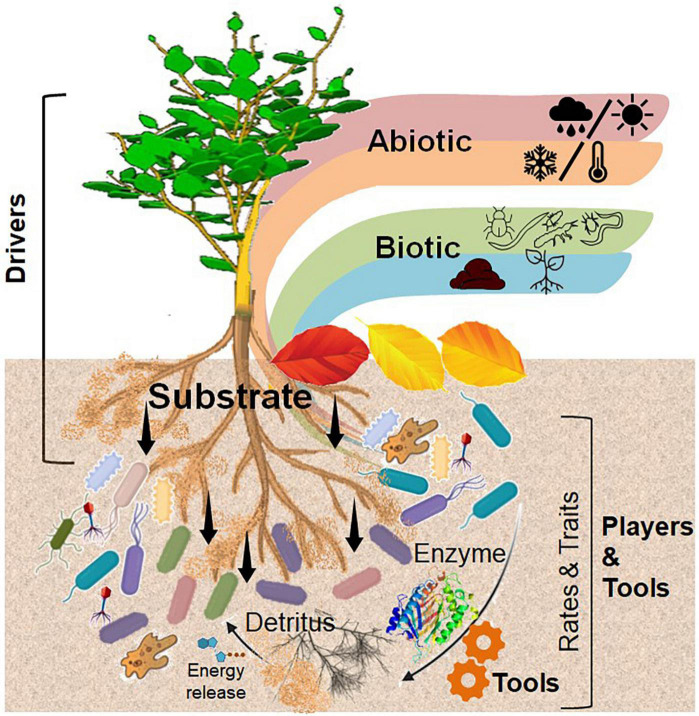
Conceptual illustration of central role of the rhizosphere in interactions with other biogeochemical interfaces. The main driver of plant–microbial interactions is an input of substrate through exudation, which is controlled by biotic and abiotic factors. Microorganisms are the most powerful players in the soil interfaces, using functional traits (e.g., the ability to produce specific extracellular enzymes) as a tool to develop a microbial life strategy, which in turn influences the rates of transformation of organic compounds in the soil. Self designed based on Song et al. (2020) (Figure 1).

Plants are the major primary producers in terrestrial ecosystems and are thus predominantly responsible for organic C input into the soil. They modulate their surrounding soil environment either *actively*, that is, producing exudates and exo-enzymes or *passively* through root and litter detritus (Kaiser et al., 2015), thus interacting with the corresponding microorganisms near the roots or of other soil interfaces [e.g., detritusphere, (bio)-pores, and aggregate surfaces]. Considering the active role of roots crossing, penetrating, and even forming aggregates, biopores, and detritus, we mainly focused on the rhizosphere and its overlap with other relevant interfaces (Figure 2).

### Rhizosphere

An essential part of C assimilated via plant primary production enters the soil through roots by a process called rhizodeposition. The growing root tip and its rhizodeposits turn the bulk soil into a rhizosphere soil with its specific physical, chemical, and biological characteristics, which convert it into a hotspot of biological activity compared to the surrounding bulk soil (Goberna et al., 2007; Reinhold-Hurek et al., 2015). Thus, specific microbial communities develop in the endosphere, rhizoplane, and rhizosphere, that is, within a few millimeters from the root surface (Edwards et al., 2015; Vidal et al., 2018) and along the growing root (Cheng et al., 2017; Schmidt et al., 2018). Continuous root growth and turnover drive the spatial distribution and transformation of primary and secondary C input into the soil. This C input is determined by a combination of factors related to root type (Swinnen et al., 1994a,b, Jahnke et al., 2009), root age, root turnover rate, and specific rhizodeposition processes (Steer and Harris, 2000; Kawasaki et al., 2016). Root growth also leads to the creation of specific interfaces between the rhizosphere and other boundaries, such as the detritusphere, (bio)-pores, or aggregate surfaces (Figure 1).

Rhizodeposits range in complexity from cells and lysates to small organic molecules. Rhizodeposits are released as a consequence of tissue turnover, sloughed-off border cells, mucilage release, or the secretion of biochemically diverse root exudates (Farrar et al., 2003; Nguyen, 2003; Jones et al., 2009). The production rate and quality of rhizodeposits are governed by plant species and even genotype (Lesuffleur et al., 2007; Mönchgesang et al., 2016), the growth rate and age of an individual plant (Gransee and Wittenmayer, 2000; Zhalnina et al., 2018), and root morphology, for example, root type or root hairs (Poirier et al., 2018; Cotton et al., 2019).

Generally, plants adapt their source–sink relationships dynamically under various abiotic-biotic environments, ensuring seed production to provide offspring and supporting growth (Smith et al., 2018). Consequently, at different time scales, C allocation, and distribution patterns in the plant body and rhizosphere vary widely in terms of allocation rates, compound variety, and quality (Brüggemann et al., 2011). For example, the net C allocation from shoot to root strongly depends on the plant functional type, with a mean value of 21% for crops and 33% for grasses (Pausch and Kuzyakov, 2018), and on plant phenological stage, with a greater allocation to roots at early plant stages (40–60% of photosynthetically-fixed C) than at the reproductive and ripening stages (less than 15%) (Swinnen et al., 1994a). According to temporal profiles, C allocation within the root system is highly dynamic and differs between the internal structures of root organs (Jahnke et al., 2009). The proportion of root C moving into the rhizosphere as rhizodeposition ranges from 1.3 to 20% of photosynthetically-fixed C for crops, trees, and perennial grasses (Jones et al., 2009; Kaiser et al., 2015; Pausch and Kuzyakov, 2018) to 20–30% for cereals and mycorrhizal plants (Jakobsen and Rosendahl, 1990; Kuzyakov and Domanski, 2000), and up to 50–60% (Lynch and Whipps, 1990; van Dam and Bouwmeester, 2016), with substantial uncertainty that is still apparent with regard to ecosystem type and climatic conditions.

Soil-plant interactions in the rhizosphere extend several millimeters from the root surface to the soil (Dazzo and Gantner, 2013). The intensity of root-soil interactions is demonstrated by pronounced distance gradients from the root surface (rhizoplane) through the rhizosphere to the bulk soil (Kuzyakov and Razavi, 2019). The formation of *chemical* gradients in the rhizosphere is governed by the input of labile root exudation, which changes their localization in accordance with root growth. Root exudation boosts the activity and modulates the community structure of soil microorganisms, thereby explaining *spatial biological* gradients in the rhizosphere (Kuzyakov and Blagodatskaya, 2015). Rhizodeposition fluctuates in space and time due to root growth and stimulates the “rhizosphere priming effect” (Cheng et al., 2014; Keiluweit et al., 2015; Nie et al., 2015), which is relatively short-term because the C from rhizodeposits is rapidly converted into microbial biomass and is partly released by microbial respiration; consequently, decomposition rates are reduced in the absence of fresh C input (De Graaff et al., 2010). Furthermore, the broad spectrum of compounds in rhizodeposits (el Zahar Haichar et al., 2014; Zhalnina et al., 2018; Pett-Ridge et al., 2020) modulates biological activities in a compound mixture-dependent manner. In particular the presence of certain sugars or secondary metabolites such as phenolic compounds can exert profound negative and positive influences on community composition and the functional potential of microorganisms (Badri et al., 2009; Chaparro et al., 2013; Cotton et al., 2019), affecting microbial growth, respiration, and decomposing activity (Chigineva et al., 2009; Zwetsloot et al., 2018).

Over time, a decrease in root exudations, for example, due to a switch from vegetative to regenerative growth (Aulakh et al., 2001; De-la-Peña et al., 2010), reduces the abundance of rhizosphere microorganisms (Chaparro et al., 2014; Schmidt and Eickhorst, 2014), ultimately leading to a downregulation of enzyme production. Therefore, the temporal-spatial shifts in rhizosphere gradients of microbial activity impact soil functions such as decomposition and nutrient mobilization (Kuzyakov and Xu, 2013; Nuccio et al., 2020). As the rhizosphere is one of the most dynamic interfaces actively developing in the local environment, the interaction of the rhizosphere with other interfaces, for example, aggregates, porosphere, and detritusphere, can essentially affect the functional traits of dominant microbial populations and the rates of microbially-mediated soil processes at these interfaces.

### Mycorrhizosphere

Mycorrhizal fungi form widespread symbiotic networks with the roots of most land plants, where the fungus delivers mineral nutrients to the mycorrhizal host plant and takes up plant sugars and lipids. Plant C flows to the soil through mycorrhizal roots, together with the external mycorrhizal mycelium, which are defined as “mycorrhizosphere” (van der Heijden et al., 2015), while the fungal hyphae as habitats for microorganisms are generally referred to as “hyphosphere” The external mycelium that may extend centimeters from the root surface to nutrient patches provides plant-derived C rapidly, in hours, to soil microorganisms (Drigo et al., 2010; Kaiser et al., 2015; Gorka et al., 2019), and this resource is used not only for growth by bacteria and fungi but also as a “priming” resource for decomposition (Fontaine et al., 2003). Priming via the mycorrhizosphere has been suggested for the widely distributed arbuscular mycorrhizal symbiosis (Cheng et al., 2012), and priming by arbuscular mycorrhizal fungi is associated with modifications to the soil microbial community composition (Herman et al., 2012; Nuccio et al., 2013; Gui et al., 2017). Consequently, arbuscular mycorrhizal symbiosis may influence different trophic levels by enhancing C allocation into the food web, stimulating N mobilization from SOM, and subsequent transfer to the host plant (Koller et al., 2013b; Hünninghaus et al., 2019). In contrast, ectomycorrhizal fungi produce extracellular enzymes and free radicals that release N from organic compounds (Lindahl and Tunlid, 2015; reviewed by Nicolás et al., 2019). By two distinct mechanisms related to litter decomposition stage and soil depth, they either suppress or stimulate decomposition (Brzostek et al., 2015; Sterkenburg et al., 2018). In topsoil with fresh litter, ectomycorrhizal competition for N decreases the decomposition rate (Averill et al., 2014), whereas in deeper soil layers with litter at later stages of decomposition, ectomycorrhizal fungi contribute to decomposition (Sterkenburg et al., 2018).

### Rhizosphere–Detritusphere Interactions

Plant photosynthates are released into the soil in the form of soluble root exudates and plant detritus (e.g., leaf litter and rhizo-detritus). Thus, rhizodeposition processes overlap with leaf litter and dead root tissue degradation processes, forming a rhizosphere–detritusphere interface. Because of the spatial and chemical heterogeneity of rhizodeposits and rhizo-detritus in soil, the microbial communities and activities in these two spheres as well as in the overlapping sphere are specific (Marschner et al., 2012; Nuccio et al., 2020). It has been shown that root activity modulates the decomposition processes in the detritusphere by altering the structure of the microbial community feeding on detritus. Distinct microbial taxa are involved in ^13^C-labeled rice straw degradation in the rhizosphere compared to bulk soil (Maarastawi et al., 2018). Essentially, less ^13^C is assimilated by microorganisms from the straw in the rhizosphere versus bulk soil, likely due to the higher availability of labile C in the rhizosphere. The availability of detritus, in turn, reduces the consumption of root exudates by the rhizosphere microbiota (Maarastawi et al., 2019), indicating that detritusphere processes modulate rhizosphere processes and demonstrate interactions between the rhizosphere-detritusphere interface. A recent study by Nuccio et al. (2020) observed higher taxonomic and functional diversity in the combined rhizosphere–detritusphere, suggesting that the co-existence of rhizosphere guilds is facilitated by niche differentiation. This observation was based on a metatranscriptomics study where the expression of genes responsible for the decomposition of different organic substrates was studied comparatively in soils that originated either from growing roots, from decaying root material, or from overlapping spheres. Spatial and temporal niche differentiation by functional genes responsible for similar functions clearly confirmed the strong redundancy of the functions in the rhizosphere and detritusphere. For example, for various functions (genome classes), the number of active genomes shared between the rhizosphere and detritusphere is 28 (housekeeping gene gyrase A, B), 32, 67, and 50% (oligosaccharide hydrolases, cellulases, and xylanase-encoding genes, respectively) of the total number of functionally active taxa (data extracted from Figure 3 in Nuccio et al., 2020). Moreover, rhizosphere organisms express genes involved in the consumption of primary C compounds as well as breakdown products, indicating that they benefit from synergistic consumption processes. Thus, such overlapping interfaces have consequences for intra- and inter-specific interactions involving soil biota. For example, changes in litter quality in the root zone alter not only bacterial community structure and function, but also cause strong feedback from bacterial grazers, thus affecting links in the soil *food webs* (Koller et al., 2013a). Therefore, successional changes in bacterial and fungal populations during plant development may lead to a corresponding succession of protist communities in the rhizosphere, which translates into less complex and dense protist networks during plant senescence (Ceja-Navarro et al., 2021).

Differences in the quality of organic matter input induce contrasting competition situations within the rhizosphere and detritusphere. In the rhizosphere, the majority of easily available organic C, such as sugars, amino acids, and carboxylic acids, are released through living roots (Jones et al., 2009). In the detritusphere, such easily degraded monomers are rapidly used up, leaving behind higher molecular weight compounds such as cellulose or lignin, thereby supporting different functional guilds (Pascault et al., 2013; Pepe-Ranney et al., 2016; Nuccio et al., 2020). Generally, root morphological properties (e.g., root hairs, fine roots, mycorrhiza) intensify the release of exudates, thus increasing microbial activity, functionality, and consequently substrate utilization, thereby stimulating rhizosphere nutrient mobilization. However, at the same time, plants take up high levels of nutrients from the rhizosphere and can thus represent a strong competitor for nutrient resources, thereby reducing microbial growth (Bonkowski et al., 2000; Blagodatskaya et al., 2014). Therefore, competition for nutrients can exist in the rhizosphere, occurring mainly between plants and other soil organisms (Kuzyakov and Xu, 2013), while competition in the detritusphere occurs primarily within or between microbial species (Esperschütz et al., 2011). Due to the contrasting quality of C sources (root-derived low molecular weight C versus more macromolecular organic compounds in detritus), the abundance and successional changes of a microbial community may differ in intensity and dynamics between the rhizosphere and detritusphere. These differences and dynamics, in turn, may be a major determinant of predatory bacteria, RNA viral, and RNA phage dynamics. The functional traits of predatory versus non-predatory bacteria reveal faster growth and much faster C assimilation rates, which are comparable to the C flow through viruses and are substantially higher than those in predatory eukaryotes (Starr et al., 2020; Hungate et al., 2021). There is evidence that changes in the structure and dynamics of multi-level trophic interactions correspond to differences in energy flow between the rhizosphere and detritusphere. Thus, both the microbial community and the community of RNA eukaryotic viruses, as well as the community of phages inhabiting the detritusphere, are more distinct in structure from bulk soil than the rhizosphere community (Starr et al., 2019). As a result, successional changes in community structure are driven to a large extent by mycoviruses and phages, bacterial predators, and by protozoan grazers, demonstrating clear temporal population dynamics and patchy spatial distribution.

### Rhizosphere–Porosphere Interactions

Rhizosphere processes are strongly influenced by interactions with the porosphere, forming a specific microbiome enriched in copiotrophic bacteria (Uksa et al., 2015; Blaser et al., 2016). Interactions with the porosphere alter the spatial expansion of the rhizosphere in soil, as root growth and architecture are affected by biopores (Han et al., 2015). Soil pores of different origins (e.g., root or earthworm-derived) serve as habitats for microorganisms, as well as conduits for chemical transport and water flow, and thus play a key role in controlling the rates of soil biochemical processes (Kravchenko et al., 2015; Negassa et al., 2015). Porosphere conditions influence microbial functioning due to the presence of roots, hyphae (Pagliai and De Nobili, 1993; Quigley et al., 2018), O_2_ levels (Keiluweit et al., 2016, 2017), or root exudate composition. Thus, it is not surprising that various groups of microorganisms are preferentially localized in pores of different sizes (Ruamps et al., 2013) or origin. C substrates localized in large pores are typically processed more rapidly than in small pores (Killham et al., 1993), and dissolved organic matter in small pores is more complex; hence, it is less decomposed than that in larger pores (Bailey et al., 2017; Toosi et al., 2017). Such a difference is not limited to size but also to the origin of the pores. For instance, *biopores* of decomposed roots or the drilosphere made by earthworms can host different varieties of microbes with distinct growth rates and efficiencies of growth (Ma et al., 2017; Hoang et al., 2020). The complexity of such systems increases even more when these spheres penetrate each other. For instance, earthworms reuse biopores of decomposed roots leaving behind the pore wall coatings or when roots grow within the drilosphere (Pagenkemper et al., 2013, 2015).

### Rhizosphere–Aggregate Interactions

Roots and associated fungal hyphae enmesh particles and release agglutinating compounds, thus building up aggregates. Soil is defined as a group of primary soil particles (and smaller aggregates) that cohere to each other more strongly than surrounding non-cohesive particles and are considered as soil structural building units (Tisdall, 1996). A hierarchy of soil aggregates ranges from macroaggregates (>250 μm) that are unstable and susceptible to soil management to the more stable microaggregates (<250 μm) (Six et al., 2004). The group of microaggregates is not homogeneous and is organized even at the smallest scale <2 μm (Totsche et al., 2018). The primary structural units of microaggregates are composed of silicates, metal oxyhydroxides, organic matter, as well as microbial debris (Chenu and Plante, 2006). The role of roots is especially relevant in the formation of 53–250 μm micro-aggregates, while the formation of smaller aggregates (<53 μm) is mainly governed by microorganisms, clay particles, and physicochemical forces (Tisdall and Oades, 1982; Rillig and Mummey, 2006; Dultz et al., 2018). Due to relatively fast root growth and its associated rhizodeposition, aggregate formation and turnover in the rhizosphere and the root-mediated shaping of aggregate surfaces are highly dynamic processes (Wang et al., 2020).

The rhizosphere is a remarkable interface, where the *aggregatosphere* interacts with the *detritusphere*, because root detritus (sloughed root cells, dead root fragments, and residues) provide a substrate for microbial metabolism. Both, microbial metabolic products and rhizodeposits form sticky polymeric substances (Redmile-Gordon et al., 2014), which are involved in the enmeshing and gluing of aggregates (Golchin et al., 1994) by binding mineral soil particles and organic fragments in the way of homoaggregation as well as in heteroaggregation (Dultz et al., 2019). The aggregate stability at the rhizosphere–detritusphere interface is directly related to biotic factors such as root biomass, microbial, macro- and micro-faunal activity, all of those being involved in the structuring of aggregate surfaces (Golchin et al., 1994) by modifying the soil biotic and abiotic environment. Furthermore, positive correlation between soil water-stable aggregates and content of extracellular polysaccharides (EPS) indicated that soil abiotic conditions such as pH and water potential are the primary controllers of both, aggregate stability and microbial EPS production (Sher et al., 2020). This results in structural and functional self-organization of the pore space, which improves microbial habitats (Young and Crawford, 2004). In turn, aggregates, as a habitat for organisms, not only organize the soil microbiome but also serve as “concurrent incubators” that provide a refuge for microbes against predation (Hemkemeyer et al., 2014; Raynaud and Nunan, 2014).

## Relevant Dynamic Drivers of Microbially-Mediated Soil Processes

### Bio-Physical Conditions

The role of roots in aggregate formation has essential implications on rhizosphere physical properties such as O_2_ diffusion, which affects both microbiome and physiological root activities. Radial root growth and shrinkage create gaps along the root surface (Carminati et al., 2011) which may serve as conduits for preferential gas transport and enhance the replenishment of O_2_ consumed by aerobic respiration in deeper soil layers (Uteau et al., 2013). Growing roots also create new pores in the rhizosphere due to water extraction, which results in intensified drying and wetting cycles (Materechera et al., 1992; Rasse et al., 2000) or in local stress concentrations forming shear cracks, thus enhancing pore network connectivity in the surrounding root (Aravena et al., 2014). Air-filled porosity below a threshold of 12–15% is not sufficient to deliver enough O_2_ for root respiration in *Zea mays*, thus reducing the rates of root elongation due to low O_2_ levels (Grable and Siemer, 1968). This requirement of maize for air-filled porosity is higher than the 10% rule-of-thumb proposed in earlier studies (Wesseling and van Wijk, 1957; Robinson, 1964; Grable, 1966); nevertheless, the ability of the root to modify its surrounding soil structure helps to circumvent this issue (Lucas et al., 2019). The action of the root modifying its environment to ensure rapid O_2_ transport (Hinsinger et al., 2009) facilitates organic matter turnover in the rhizosphere (Jones and Hinsinger, 2008) because of intensified microbial activity compared to bulk soil (Nunan et al., 2003). Although most of the soil’s respiratory activity (microbial and root respiration) occurs in the rhizosphere (Kuzyakov, 2002; Raynaud, 2010), only a small number of studies have described the spatial distribution of O_2_ in structured aerobic rhizosphere soil, considering the water regime. A water content of approximately 30–40% of soil field capacity generally ensures high respiration rates, highlighting the importance of assessing moisture levels when estimating the required O_2_ supply (Balogh et al., 2011). At high moisture levels, microorganisms accelerate their metabolism, and at the same time, more pores are blocked by water bridges, limiting oxygen diffusion. Reduced redox potential at root surfaces due to high O_2_ consumption rates (Fischer et al., 1989) forms a gradient of oxygen concentrations, which ranges from very low at the root surface to the average soil concentration at a distance of approximately 15 mm (Keiluweit et al., 2015). Gradients of redox potential are most pronounced at the root tips, extending up to 3 mm from the root surface. Oxygen limitation can be detected at matric potentials exceeding a threshold value of approximately -3 kPa up to field capacity, showing a clear gradient while approaching the root’s surface that sharply decreases at a distance of 2–3 mm from the root surface (Uteau et al., 2015). Modeled O_2_ consumption in the rhizosphere demonstrates dynamic microbiome responses to O_2_ supply and the importance of soil structure around roots (Uteau et al., 2015).

### Biochemical Conditions

Up to one-third of photosynthates allocated to roots is released to the soil, i.e., is “lost” by the plant (Pierret et al., 2007). Such losses through rhizodeposition (Lynch and Whipps, 1990) and release of protons (Ayres et al., 2009; Andrianarisoa et al., 2010; Cesarz et al., 2013) serve as plant investments to develop and modify the physical and biochemical properties of the rhizosphere environment to improve nutrient uptake (Augusto et al., 2002). Root exudates in the form of low molecular weight solutes strongly affect nutrient solubility, microbial activities, and the turnover of microbial biomass, as well as interactions between plants (Helal and Sauerbeck, 1986; Bertin et al., 2003; Vives-Peris et al., 2020), and the production of extracellular enzymes (Asmar et al., 1994), thereby indirectly influencing nutrient availability (Grayston et al., 1997; Hamilton and Frank, 2001; Herman et al., 2006; Landi et al., 2006). Roots can bypass the surrounding soil volume by self-regulation via the production of root hairs and exudates, by which more photosynthetic resources are allocated below-ground (Pages, 2002). Furthermore, the release of signaling molecules such as abscisic acid present in root exudates (Hartung, 2010) promotes the selection of particular microbial taxa within the vicinity of the root system (Marschner et al., 2004), allowing efficient complementary functioning of roots with microorganisms for nutrient mobilization. The release of H^+^ by roots into soils is one of the dominant mechanisms of plant nutrient mobilization and maintenance of a proper electrochemical potential in the rhizosphere (Marschner and Rengel, 2012). Among various plants, legumes strongly acidify rhizosphere soil (Israel and Jackson, 1978; Haynes, 1983), while certain other plants (e.g., most cereals) release OH^–^ ions via the roots (Youssef et al., 1989). Overall, the ability of plant species to influence rhizosphere pH depends on the initial soil pH as well as N fertilization (Kuzyakov and Razavi, 2019).

### Trophic Interactions

An additional relevant driver of microbial processes is interactions with soil organisms of higher trophic levels (Scheu et al., 2005). For example, rhizobacteria are top-down regulated by grazers, particularly by protists (Clarholm, 1985; Bonkowski, 2004). Grazing strongly affects the composition and functional evolution of microbial communities and fosters C and N mineralization from detritus for plant uptake (Alphei et al., 1996; Geisen et al., 2018). These mineralization processes depend on spatial distribution, size, and detritus quality (Bonkowski et al., 2000; Koller et al., 2013b). During decomposition of labile and recalcitrant C fractions of detritus, protist communities themselves undergo temporal succession at fine spatial and temporal scales (Hünninghaus et al., 2017). Microbial processes are also shaped by interactions with soil fauna (Bonkowski et al., 2000). For example, density-dependent and selective feeding by fungivore soil fauna affects the balance between mycorrhizal and saprotrophic fungi, nutrient mobilization, and thus plant performance (Klironomos and Ursic, 1998; Tiunov and Scheu, 2005). However, soil faunal activity affects physical soil structures, such as pores and microhabitats (Maraun et al., 1999; Eisenhauer et al., 2010). Therefore, faunal activity imposes spatial restrictions on soil organisms to sense and access food resources that shape trophic interactions (Erktan et al., 2020). The given examples above highlight the temporal and spatial complexity of multitrophic interactions as drivers of microbial processes. In turn, higher trophic level organisms, such as bacterial-feeding nematodes or protists, can provide strong feed-back in terms of bacterial respiration and nutrient mobilization, with the latter process being directly relevant for plant growth (Bonkowski, 2004; Brüggemann et al., 2011).

## Relevant Scales for Process Localization

The ecological relevance of a soil process (gaseous emission, C sequestration, nutrient cycling, or leaching) is generally determined at the macro-scale, for example, on the landscape or soil profile level. Such approaches are important for global budget estimates. Understanding the mechanisms and spatial distribution of these processes requires, however, more precise mesocosm studies, while a shift to the micro- and even to the nanoscale is necessary to identify links between rates and distinct local processes in soil microhabitats.

Recent progress in process visualization at the mesoscale (root scale) was achieved using novel microsensor techniques and soil zymography. These approaches enable the monitoring of the *two*-dimensional distribution of soil properties, such as pH or oxygen concentration (Blossfeld and Gansert, 2007; Blossfeld et al., 2013) and the intensity of SOM decomposition (e.g., by CO_2_ and hydrolytic enzyme activities). Zymography coupled with X-ray CT is very promising for the 3D reconstruction of enzymatic processes within soil pore spaces, in particular, if the integration of 2D chemical imaging (in this case zymography) and 3D (μ)X-ray CT is further coupled with the modeling of pore-scale processes (Roose et al., 2016). For example, there are spatially explicit models for nutrient uptake by roots and root hairs based on addressStreetX-ray CT (Daly et al., 2016) that could be coupled with the 3D reconstruction of enzymatic processes for a comprehensive insight into enzyme-driven nutrient mobilization in the rhizosphere. A 4D visualization of dynamic developments of a process within the soil volume as well as a shift in zymography from the meso- to the microscopic scale remains a challenge. Such a challenge can be realized through the coupled visualization of soil processes and the estimation of localized process rates. This requires a methodology that considers biotic and abiotic drivers, functionally and phylogenetically diverse players, and multiple resolution scales of soil biochemical processes.

## Relevant Approaches for Process Localization

Existing and newly developed methods for the determination of localized process rates in soil can be differentiated into three groups based on (i) *destructive* sampling disturbing soil microcosms, (ii) non-destructive imaging *in situ* techniques, and (iii) prediction by modeling.

### Destructive Approaches

Techniques to identify microorganisms are currently mostly based on DNA sequencing approaches, as DNA has high information content regarding taxonomy (Table 1). Metagenomic sequencing can be used to obtain information concerning the broad functional potential of the microbiome when DNA sequencing is not limited to specific PCR-amplified phylogenetic or functional markers. To identify or quantify more specifically active decomposers, different methods have been developed, including meta-transcriptomics (Antunes et al., 2016; Yergeau et al., 2018; Bei et al., 2019; Nuccio et al., 2020), nanoscale secondary ion mass spectrometry (nanoSIMS) (Pett-Ridge and Firestone, 2017; Vidal et al., 2018), stable isotope probing of DNA, RNA, or PLFAs (Hannula et al., 2012; Hünninghaus et al., 2019; Maarastawi et al., 2019) and other techniques that relate abundance to metabolically active microbial consortia (Baldrian et al., 2012; Emerson et al., 2017). To predict process rates, focus on the quantitative parameters of a process rather than the mere structure of the microbial community is needed (Malik et al., 2020), which can be provided by measuring expression rates of genes involved in specific processes, determined by RT-qPCR to quantify expression of specific functional genes, or by metatranscriptomics, in which transcript numbers can be taken as a proxy for gene expression rates. However, it must be kept in mind that gene expression levels do not necessarily correlate with protein abundance, enzyme activity, or respiratory-based physiological approaches (Nannipieri et al., 2003). Recently, qSIP was proposed as an approach to quantify the metabolic activity of all specific groups of microorganisms that contribute to the substrate conversion process (Hungate et al., 2015; Papp et al., 2020).

**TABLE 1:** Comparison of processes monitored by various approaches.

Method Processes/traits	Predictive	Destructive	Non-destructive	Precise micro-sampling
Root exudation	Exporter gene presence	Gene expression	Marker construct or knockout, autoradiography	Microdissection
Root/microbial traits and functions	Geochip, functional marker genes, specific marker genes, qPCR	RT-qPCR, qSIP, molecular biomarkers	Scanning transmission X-ray microscopy	NanoSIMS, FISH, X-ray microscopy
pH	Correlative statistics	Suspension	Optodes	Microsensors
P solubilization	*pho* genes presence	Phosphatases & phytases	Zymography	Hotspots sampling
Respiration CO_2_, O_2_	Mechanistic Models based on local difussivities	Basal respiration	Optodes, Clark-type-based glas microelectrodes, dyes, soil cores	Ion beam slicing (down to micrometer thickness)
C and N transformation	Functional marker genes	Enzymatic approaches	Zymography	Hotspots sampling

*References used for this table can be found in the Supplementary Material.*

#### Methodological Constraints

Whereas comparisons of values obtained within specific studies can provide valuable information concerning the performance of individual taxa under specific conditions and over a given period of time, comparison of microbial growth rates between studies based on molecular and physiological approaches reveals certain methodological constraints. Microbial-specific growth rates determined by SIGR in contrasting soil microhabitats (Figure 1) are comparable, but are reasonably slower than exponential growth of *E. coli* in pure culture (0.2–1.1 h^–1^, You et al., 2013). The specific bacterial growth rates obtained by isotope trace labeling (e.g., 0.33 h^–1^, Meisner et al., 2013) are in good agreement with SIGR (Figure 1). However, growth rates estimated by ^18^O qSIP in soils without substrate input (0.0002–0.001 h^–1^, data extracted from Figure 4 in Morrissey et al., 2018) and after glucose addition (0.0001–0.0065 h^–1^, data extracted from Figure 2 in Li et al., 2019) were 2–3 orders of magnitude lower than microbial growth as assessed by SIGR (Figure 1). This underestimation of growth rates by ^18^O qSIP occurred despite the calculations in a study by Li et al. (2019), who considered that only 60% of the oxygen in DNA comes from water (Koch and Munks, 2018). The rates obtained for growth on glucose by qSIP were several orders of magnitude slower than those obtained by SIGR. First, the growth rate calculations by qSIP were based on an incorrect assumption of steady state after glucose addition, neglecting that microorganisms are not in a steady state during growth. Second, the application of qSIP one week after substrate input is too late compared to exponential growth usually occurring within 20–48 h after soil activation with substrate (see e.g., Meisner et al., 2013; Loeppmann et al., 2020). Therefore, microbial growth rates determined 7 days after glucose addition mirror substrate-induced *successional changes* and *substrate re-utilization* rather than bacterial growth rates. Fungal and bacterial growth rates are very dynamic and can rise or fall up to 7–10 times within one week after substrate addition; thus, a shift of several days can occur between the peak values for bacterial and fungal growth (Nannipieri et al., 1978; Silva-Sánchez et al., 2019). Thus, the task of relating gene phenotypic traits to *in situ* growth rates still remains a challenge and requires consideration of microbial physiology in experimental designs.

The goal of quantifying the incorporation of a stable isotope label in specific groups of microorganisms has also been achieved by combining microarray analysis with nanoSIMS (Mayali et al., 2011). Incorporation of isotopically-labeled substrates into microbial biomolecules serves as a quantitative proxy for microbial activity, contributing to the decomposition of SOM or SOM components. However, this approach requires a degree of caution when comparing quantitative data involving studies using different time intervals and various types of biomolecules, because incorporation of isotope labels is affected by the turnover times of these biomolecules; for example, nucleic acids are more rapidly labeled relative to PLFAs and membrane lipids (Malik et al., 2015).

### Non-destructive Approaches

The development of new approaches and concepts is not evenly distributed among the interfaces (hotspots); in particular, modern viewpoints are mostly presented for the rhizosphere (Table 1). For instance, most *in situ* techniques have been adapted for imaging rhizosphere properties and processes (Oburger and Schmidt, 2016). These approaches include i) optodes for measurement of CO_2_, pH, O_2_ (Blossfeld et al., 2011; Rudolph et al., 2013), (ii) sensitive gels (pH indicators (Römheld, 1986), (iii) zymography for enzyme activity (Spohn and Kuzyakov, 2013; Razavi et al., 2019), (iv) DGT gel (Diffusive Gradient in Thin-films) for elements (Fresno et al., 2017), and (v) imaging of radioactive isotopes: ^14^C (Pausch and Kuzyakov, 2011), ^33^P, ^32^P; ^40^Ca for nutrients and neutron imaging for water (Carminati and Vetterlein, 2013), and enable visualization of spatio-temporal patterns of rhizosphere properties and rhizosphere processes (Kuzyakov and Razavi, 2019). Such novel techniques have revealed a multiscale (time and space) examination of plant-microbiome interactions and their functionality (Baveye et al., 2018).

#### Methodological Constraints

Although visualization techniques enable quantitative estimates based on calibration, many still remain qualitative or semi-quantitative and do not exhibit consistent correspondence with the process rates and activity obtained by destructive sampling. For example, for approaches based on the application of sensor gels or membranes to the soil surface (i.e., optodes or zymography), essential methodological uncertainties occur related to the diffusion of targeted colored or fluorescent molecules (substrates or products of reactions) between the soil and membrane as well as within the membrane (Guber et al., 2018). Possible solutions for this problem could be a combination of activity hotspot localization by zymography with precise destructive micro-sampling after visualization (Tian et al., 2020) or visualization of the processes at the microscopic scale, avoiding attachment of artificial sensors or membranes (Table 1). Certain disagreements also occur involving molecular approaches (identifying plant and microbial traits by functional genes) and estimation of process rates, for example, by enzymatic activity (Kumar et al., 2017; Nilsson et al., 2019). Such disagreement confirms that gene existence does not necessarily reflect the activity of the corresponding protein (Nannipieri et al., 2018).

Hence, a quantitative estimation of process rates and the magnitude of changes in pools and fluxes is necessary at interfaces such as the rhizosphere to clarify, for instance, how inoculants modulate the resident microbiome, how pathogenic attack affects the activity of the complex microbiota of hotspots, how grazing activities by protists, nematodes, or bacteriophages control the extinction of species, and how the rhizosphere microbiome responds to abiotic stresses (e.g., salinity, drought, heat). This remains a challenging task, considering the diversity of C compounds in the rhizosphere and the challenges regarding their analysis (van Dam and Bouwmeester, 2016; Oburger and Jones, 2018).

### Prediction Based on Statistical Analysis of Process Locations

Although a range of rhizosphere process-related parameters (e.g., pH, CO_2_, P, Mn content, and enzyme activity) are satisfactorily visualized in 2D by application of sensor membranes to the root–soil interface (Blossfeld and Gansert, 2007; Blossfeld et al., 2013), the localization of these parameters within the soil volume requires undesirable destructive sampling (Table 1). From CT-based 3D root localization within the soil domain, the probability distribution of the distance of a randomly selected location to the nearest root (Schlüter et al., 2018) can be computed, which potentially enables the development of a probabilistic 3D model to co-localize spatially-resolved arrays of rhizosphere-relevant parameters and the 3D architecture of root systems, for example, using Gaussian random fields (Blossfeld and Gansert, 2007; Blossfeld et al., 2013; Hristopulos, 2020). Such a model is easily extensible to account for local heterogeneity in the soil as well as topological and morphological properties of root architecture, such as branching, root tips, and root age. Moreover, spatial resolution of the predicted parameters can be defined by the underlying 2D measurements, thus enabling the investigation of various soil interfaces, as outlined above. By co-registration of MRI–PET (Jahnke et al., 2009), a 3D non-invasive analysis of plant structures and recently fixed C-transport processes within a root structure that may change in response to genomic, developmental, or environmental challenges may be established.

A stereological technique based on root architecture models such as CPlantBox (Schnepf et al., 2018a,b) provides a further promising perspective to overcome the need for expensive 3D imaging of plant roots, combining extensive model-based simulation of virtual root systems in 3D with machine learning methods. Thus, the spatially-resolved distribution of processes in the rhizosphere and other soil interfaces can be simulated in the 3D soil surrounding a plant root, using 2D measurements only.

## Conclusion and Outlook: Emergent Properties of Microbial Activity in Soil

Traditionally, total microbial biomass, potential enzyme activities, substrate-induced respiration, and organic matter content in a given volume of soil have been used to predict decomposition activity and to model the fate of organic matter. To assess how the microscale generates macroscopic behavior, the so-called emergent properties, microscale heterogeneity, dynamics of substrate properties, and microbial activities need to be taken into account (Baveye et al., 2018). This aim is multidisciplinary and extremely challenging. It is necessary to link the spatial distribution of SOM (Peth et al., 2014; Müller et al., 2016; Rawlins et al., 2016) with its combined biophysical and biochemical properties, as well as with decomposer microorganisms and their respective traits and activities in the contexts of space and time (Baveye et al., 2018). Promising techniques that consider soil micro-heterogeneity are reproducible systems that mimic the soil and can be used for hypothesis testing (Tecon and Or, 2017). Novel characterization techniques are increasingly being employed to systematically track the characteristics of organic C conversion at the soil micro-interface (Table 1).

The transformation process of organic matter and its influencing factors are discussed at the scale of micro-ecological systems. Progress in near-edge X-ray absorption fine structure spectroscopy (NEXAFS), scanning transmission X-ray microscopy (STXM), X-ray absorption spectroscopy, micro-fluorescence spectroscopy, and nanoSIMS, as well as combined STXM-NanoSIMS (Keiluweit et al., 2012; Remusat et al., 2012), applied to soil thin sections, have revealed distinct spatial heterogeneity in the chemical composition of soils over minute distances (Lehmann et al., 2005; Mueller et al., 2013). Pulse-labeling experiments in combination with NanoSIMS enable tracing of the uptake, storage, and translocation of stable isotopes (Vidal et al., 2018). The development of novel detection technologies, such as NEXAFS and X-ray photoelectron spectroscopy (XPS) during the last decades, has greatly enriched our understanding of the microscopic distribution characteristics of SOM (Amelung et al., 2002). XPS was successfully adapted to determine the chemical composition of SOMs occluded in different aggregate size fractions. In addition, the spatial distribution of elements at a resolution of < 3 μm can be mapped in selected regions of coatings, mineral-organic associations, and aggregates using electron probe microanalysis (EPMA). Significant advances related to molecular markers and detection sensitivity now enable better detection of specific bacteria in soils and their spatial distribution at the micrometer scale to be determined in thin sections (Eickhorst and Tippkötter, 2008; Castorena et al., 2016). All of this information can, in principle, be combined and translated into 3D distributions using recently developed statistical algorithms.

To conclude, this review suggests a conceptual view emphasizing the central role of the rhizosphere in interactions with other biogeochemical interfaces. The main drivers of plant–microbial interactions, such as substrate input through exudation and rhizodeposition, and physico-chemical conditions (e.g., proton release and oxygen diffusion and transport) are already subject to intensive research. In contrast, the driving role of trophic interactions within and between interfaces, including competition for nutrients and successional dynamics, requires more specific studies involving both higher and lower trophic levels (e.g., protists, predatory bacteria, and mycoviruses). According to our concept, microorganisms are not the drivers, but they are the most abundant and powerful players in the soil interfaces because of the great diversity and specificity of genes encoding similar functions. The combination of phylogenic specificity and functional redundancy ensures the sustainability of soil microbial communities by the use of functional traits (e.g., the ability to produce specific extracellular enzymes, rapid or slow growth, and efficiency of metabolic pathways) as a tool to develop a microbial life strategy, which in turn affects the rates of transformation of organic compounds in soil. Thus, taxa with life strategies best adapted to the environment become dominant and alter the structure of the active microbial community. This self-regulatory mechanism maintains metabolic activity by the microbial community during the successional decomposition of organic substrates entering the soil. However, the rates of substrate decomposition are dependent on the functional traits of dominant taxa and microbial life strategy, which in turn are selected according to substrate quality and local environmental constraints, for example, water and nutrient availability. The rapid development of instrumental and molecular techniques has fueled attempts to reconsider the concepts of microbial life strategies with the goal of specifying functional groups according to their ecological relevance. This requires the identification and estimation of intrinsic traits by microbial physiology or phenotypic traits at the functional gene level. The quantitative definition of functional traits based on genetic and isotopic approaches is very promising, but demands further development with caution regarding the relevant resolution time and type of biomarker. Additional technique development is needed for ground-truth measurements of microbial growth in soil, linking physiological and molecular approaches. Therefore, the current challenge in modern ecology is the further development of cutting-edge methodologies for precise localization of biochemical processes, considering interactions within and between soil interfaces, as well as identifying and linking functional traits of plants and microbial populations that contribute to the rates of soil processes relevant at the ecosystem level.

## Author Contributions

EB, SS, and BR developed the concept and all the co-authors contributed to the writing and structured the manuscript. All authors contributed to the article and approved the submitted version.

## Conflict of Interest

RK was employed by the company Forschungszentrum Jülich GmbH. The remaining authors declare that the research was conducted in the absence of any commercial or financial relationships that could be construed as a potential conflict of interest.

## Publisher’s Note

All claims expressed in this article are solely those of the authors and do not necessarily represent those of their affiliated organizations, or those of the publisher, the editors and the reviewers. Any product that may be evaluated in this article, or claim that may be made by its manufacturer, is not guaranteed or endorsed by the publisher.
