# Supplementary material for: Bridging Microbial Functional Traits With Localized Process Rates at Soil Interfaces
Source: Front Microbiol. 2021 Oct 28;12:625697. doi: 10.3389/fmicb.2021.625697 (PMC8581545; doi:10.3389/fmicb.2021.625697)
Supplement: Supplementary file 1 [file Data_Sheet_1.DOCX]

**Reference List to the sources of Fig. 1 and Table 1**

Ahmed, M. A., Sanaullah, M., Blagodatskaya, E., Mason-Jones, K., Jawad, H., Kuzyakov, Y., & Dippold, M. A. (2018). Soil microorganisms exhibit enzymatic and priming response to root mucilage under drought. *Soil Biology and Biochemistry*, *116*, 410-418.

Awad, Y. M., Blagodatskaya, E., Ok, Y. S., & Kuzyakov, Y. (2012). Effects of polyacrylamide, biopolymer, and biochar on decomposition of soil organic matter and plant residues as determined by 14C and enzyme activities. *European Journal of Soil Biology*, *48*, 1-10.

Baldrian, P., & Valášková, V. (2008). Degradation of cellulose by basidiomycetous fungi. *FEMS microbiology reviews*, *32*(3), 501-521.

Baldrian, P., Kolařík, M., Štursová, M., Kopecký, J., Valášková, V., Větrovský, T., ... & Voříšková, J. (2012). Active and total microbial communities in forest soil are largely different and highly stratified during decomposition. *The ISME journal*, *6*(2), 248-258.

Baldrian, P. (2004). Purification and characterization of laccase from the white-rot fungus Daedalea quercina and decolorization of synthetic dyes by the enzyme. *Applied Microbiology and Biotechnology*, *63*(5), 560-563.

Blagodatsky, S. A., Blagodatskaya, Е. V., Anderson, T. H., & Weigel, H. J. (2004). Kinetics of substrate induced respiration of bulk soil and rhizosphere microorganisms at different levels of atmospheric CO2 and N fertilization. *EUROSOIL: Freiburg/Breisgau*, 04-12.

Blagodatsky, S. A., Blagodatskaya, E. V., & Rozanova, L. N. (1994). Kinetics and strategy of microbial-growth in Chernozemic soil affected by different long-term fertilization. *Microbiology*, *63*(2), 165-170.

Blagodatsky, S. A., Blagodatskaya, E. V., Anderson, T. H., & Weigel, H. J. (2006). Kinetics of the respiratory response of the soil and rhizosphere microbial communities in a field experiment with an elevated concentration of atmospheric CO 2. *Eurasian Soil Science*, *39*(3), 290-297.

Blagodatskaya, E., Littschwager, J., Lauerer, M., & Kuzyakov, Y. (2010). Growth rates of rhizosphere microorganisms depend on competitive abilities of plants and N supply. *Plant Biosystems*, *144*(2), 408-413.

Blagodatskaya, E. V., & Anderson, T. H. (1998). Interactive effects of pH and substrate quality on the fungal-to-bacterial ratio and qCO2 of microbial communities in forest soils. *Soil Biology and Biochemistry*, *30*(10-11), 1269-1274.

Blagodatskaya, E. V., Blagodatsky, S. A., Anderson, T. H., & Kuzyakov, Y. (2007). Priming effects in Chernozem induced by glucose and N in relation to microbial growth strategies. *applied soil ecology*, *37*(1-2), 95-105.

Blagodatskaya, E. V., Blagodatsky, S. A., Anderson, T. H., & Kuzyakov, Y. (2009). Contrasting effects of glucose, living roots and maize straw on microbial growth kinetics and substrate availability in soil. *European Journal of Soil Science*, *60*(2), 186-197.

Blagodatskaya, E., Blagodatsky, S., Anderson, T. H., & Kuzyakov, Y. (2014). Microbial growth and carbon use efficiency in the rhizosphere and root-free soil. *PloS one*, *9*(4), e93282.

Blagodatskaya, E., Khomyakov, N., Myachina, O., Bogomolova, I., Blagodatsky, S., & Kuzyakov, Y. (2014). Microbial interactions affect sources of priming induced by cellulose. *Soil Biology and Biochemistry*, *74*, 39-49.

Chen, R., Senbayram, M., Blagodatsky, S., Myachina, O., Dittert, K., Lin, X., ... & Kuzyakov, Y. (2014). Soil C and N availability determine the priming effect: microbial N mining and stoichiometric decomposition theories. *Global change biology*, *20*(7), 2356-2367.

Chowdhury, S., Lange, M., Malik, A. A., Goodall, T., Huang, J., Griffiths, R. I., & Gleixner, G. (2020). Nutrient Source and Mycorrhizal Association jointly alters Soil Microbial Communities that shape Plant-Rhizosphere-Soil Carbon-Nutrient Flows. *bioRxiv*.

Dorodnikov, M., Blagodatskaya, E., Blagodatsky, S., Marhan, S., Fangmeier, A., & Kuzyakov, Y. (2009). Stimulation of microbial extracellular enzyme activities by elevated CO2 depends on soil aggregate size. *Global Change Biology*, *15*(6), 1603-1614.

Dorodnikov, M., Blagodatskaya, E., Blagodatsky, S., Marhan, S., Fangmeier, A., & Kuzyakov, Y. Stimulation of microbial enzyme activities by elevated atmospheric CO2 depends on soil aggregates size. *FACULTY AGRICULTURAL SCIENCES*, 105.

Hoang, D. T., Razavi, B. S., Kuzyakov, Y., & Blagodatskaya, E. (2016). Earthworm burrows: kinetics and spatial distribution of enzymes of C-, N-and P-cycles. *Soil Biology and Biochemistry*, *99*, 94-103.

Hoang, D. T., Pausch, J., Razavi, B. S., Kuzyakova, I., Banfield, C. C., & Kuzyakov, Y. (2016). Hotspots of microbial activity induced by earthworm burrows, old root channels, and their combination in subsoil. *Biology and Fertility of Soils*, *52*(8), 1105-1119.

Hoang, D. T. T., Maranguit, D., Kuzyakov, Y., & Razavi, B. S. (2020). Accelerated microbial activity, turnover and efficiency in the drilosphere is depth dependent. *Soil Biology and Biochemistry*, 107852.

Kumar, A., Shahbaz, M., Blagodatskaya, E., Kuzyakov, Y., & Pausch, J. (2018). Maize phenology alters the distribution of enzyme activities in soil: Field estimates. *Applied Soil Ecology*, *125*, 233-239.

Kumar, A., Dorodnikov, M., Splettstößer, T., Kuzyakov, Y., & Pausch, J. (2017). Effects of maize roots on aggregate stability and enzyme activities in soil. *Geoderma*, *306*, 50-57.

Kravchenko, A. N., Guber, A. K., Razavi, B. S., Koestel, J., Quigley, M. Y., Robertson, G. P., & Kuzyakov, Y. (2019). Microbial spatial footprint as a driver of soil carbon stabilization. *Nature communications*, *10*(1), 1-10.

Kumar, A., Phillips, R. P., Scheibe, A., Klink, S., & Pausch, J. (2020). Organic matter priming by invasive plants depends on dominant mycorrhizal association. *Soil Biology and Biochemistry*, *140*, 107645.

Loeppmann, S., Blagodatskaya, E., Pausch, J., & Kuzyakov, Y. (2016). Substrate quality affects kinetics and catalytic efficiency of exo-enzymes in rhizosphere and detritusphere. *Soil Biology and Biochemistry*, *92*, 111-118.

Loeppmann, S., Blagodatskaya, E., Pausch, J., & Kuzyakov, Y. (2016). Enzyme properties down the soil profile-A matter of substrate quality in rhizosphere and detritusphere. *Soil Biology and Biochemistry*, *103*, 274-283.

Loeppmann, S., Semenov, M., Blagodatskaya, E., & Kuzyakov, Y. (2016). Substrate quality affects microbial‐and enzyme activities in rooted soil. *Journal of Plant Nutrition and Soil Science*, *179*(1), 39-47.

Loeppmann, S., Semenov, M., Kuzyakov, Y., & Blagodatskaya, E. (2018). Shift from dormancy to microbial growth revealed by RNA: DNA ratio. *Ecological Indicators*, *85*, 603-612.

López-Mondéjar, R., Tláskal, V., Větrovský, T., Štursová, M., Toscan, R., da Rocha, U. N., & Baldrian, P. (2020). Metagenomics and stable isotope probing reveal the complementary contribution of fungal and bacterial communities in the recycling of dead biomass in forest soil. *Soil Biology and Biochemistry*, *148*, 107875.

Malik, A., Blagodatskaya, E., & Gleixner, G. (2013). Soil microbial carbon turnover decreases with increasing molecular size. *Soil Biology and Biochemistry*, *62*, 115-118.

Malik, A. A., Dannert, H., Griffiths, R. I., Thomson, B. C., & Gleixner, G. (2015). Rhizosphere bacterial carbon turnover is higher in nucleic acids than membrane lipids: implications for understanding soil carbon cycling. *Frontiers in Microbiology*, *6*, 268.

Malik, A. A., Swenson, T., Weihe, C., Morrison, E. W., Martiny, J. B., Brodie, E. L., ... & Allison, S. D. (2020). Drought and plant litter chemistry alter microbial gene expression and metabolite production. *The ISME Journal*, 1-12.

Ma, X., Zarebanadkouki, M., Kuzyakov, Y., Blagodatskaya, E., Pausch, J., & Razavi, B. S. (2018). Spatial patterns of enzyme activities in the rhizosphere: Effects of root hairs and root radius. *Soil Biology and Biochemistry*, *118*, 69-78.

Ma, X., Razavi, B. S., Holz, M., Blagodatskaya, E., & Kuzyakov, Y. (2017). Warming increases hotspot areas of enzyme activity and shortens the duration of hot moments in the root-detritusphere. *Soil Biology and Biochemistry*, *107*, 226-233.

Ma, X., Liu, Y., Zarebanadkouki, M., Razavi, B. S., Blagodatskaya, E., & Kuzyakov, Y. (2018). Spatiotemporal patterns of enzyme activities in the rhizosphere: effects of plant growth and root morphology. *Biology and Fertility of Soils*, *54*(7), 819-828.

Ma, X., Mason-Jones, K., Liu, Y., Blagodatskaya, E., Kuzyakov, Y., Guber, A., ... & Razavi, B. S. (2019). Coupling zymography with pH mapping reveals a shift in lupine phosphorus acquisition strategy driven by cluster roots. *Soil Biology and Biochemistry*, *135*, 420-428.

Mganga, K. Z., Razavi, B. S., & Kuzyakov, Y. (2015). Microbial and enzymes response to nutrient additions in soils of Mt. Kilimanjaro region depending on land use. *European Journal of Soil Biology*, *69*, 33-40.

Qiao, N., Xu, X., Hu, Y., Blagodatskaya, E., Liu, Y., Schaefer, D., & Kuzyakov, Y. (2016). Carbon and nitrogen additions induce distinct priming effects along an organic-matter decay continuum. *Scientific Reports*, *6*, 19865.

Razavi, B. S., Hoang, D. T., Blagodatskaya, E., & Kuzyakov, Y. (2017). Mapping the footprint of nematodes in the rhizosphere: cluster root formation and spatial distribution of enzyme activities. *Soil Biology and Biochemistry*, *115*, 213-220.

Razavi, B. S., Blagodatskaya, E., & Kuzyakov, Y. (2015). Nonlinear temperature sensitivity of enzyme kinetics explains canceling effect—a case study on loamy haplic Luvisol. *Frontiers in microbiology*, *6*, 1126.

Sanaullah, M., Blagodatskaya, E., Chabbi, A., Rumpel, C., & Kuzyakov, Y. (2011). Drought effects on microbial biomass and enzyme activities in the rhizosphere of grasses depend on plant community composition. *Applied Soil Ecology*, *48*(1), 38-44.

Sanaullah, M., Chabbi, A., Maron, P. A., Baumann, K., Tardy, V., Blagodatskaya, E., ... & Rumpel, C. (2016). How do microbial communities in top-and subsoil respond to root litter addition under field conditions?. *Soil Biology and Biochemistry*, *103*, 28-38.

Shahbaz, M., Kumar, A., Kuzyakov, Y., Börjesson, G., & Blagodatskaya, E. (2018). Interactive priming effect of labile carbon and crop residues on SOM depends on residue decomposition stage: Three-source partitioning to evaluate mechanisms. *Soil Biology and Biochemistry*, *126*, 179-190.

Shahbaz, M., Kuzyakov, Y., Sanaullah, M., Heitkamp, F., Zelenev, V., Kumar, A., & Blagodatskaya, E. (2017). Microbial decomposition of soil organic matter is mediated by quality and quantity of crop residues: mechanisms and thresholds. *Biology and Fertility of Soils*, *53*(3), 287-301.

Tian, P., Razavi, B. S., Zhang, X., Wang, Q., & Blagodatskaya, E. (2020). Microbial growth and enzyme kinetics in rhizosphere hotspots are modulated by soil organics and nutrient availability. *Soil Biology and Biochemistry*, *141*, 107662.

Tian, J., Pausch, J., Yu, G., Blagodatskaya, E., & Kuzyakov, Y. (2016). Aggregate size and glucose level affect priming sources: a three-source-partitioning study. *Soil Biology and Biochemistry*, *97*, 199-210.

Tian, J., Pausch, J., Yu, G., Blagodatskaya, E., Gao, Y., & Kuzyakov, Y. (2015). Aggregate size and their disruption affect 14C-labeled glucose mineralization and priming effect. *Applied Soil Ecology*, *90*, 1-10.

Tian, J., Pausch, J., Yu, G., Blagodatskaya, E., & Kuzyakov, Y. (2016). Aggregate size and glucose level affect priming sources: a three-source-partitioning study. *Soil Biology and Biochemistry*, *97*, 199-210.

Wutzler, T., Blagodatsky, S. A., Blagodatskaya, E., & Kuzyakov, Y. (2012). Soil microbial biomass and its activity estimated by kinetic respiration analysis–Statistical guidelines. *Soil Biology and Biochemistry*, *45*, 102-112.

Wei, X., Hu, Y., Razavi, B. S., Zhou, J., Shen, J., Nannipieri, P., ... & Ge, T. (2019). Rare taxa of alkaline phosphomonoesterase-harboring microorganisms mediate soil phosphorus mineralization. *Soil Biology and Biochemistry*, *131*, 62-70.

Wei, X., Razavi, B. S., Hu, Y., Xu, X., Zhu, Z., Liu, Y., ... & Ge, T. (2019). C/P stoichiometry of dying rice root defines the spatial distribution and dynamics of enzyme activities in root-detritusphere. *Biology and fertility of soils*, *55*(3), 251-263.

Wei, L., Razavi, B. S., Wang, W., Zhu, Z., Liu, S., Wu, J., ... & Ge, T. (2019). Labile carbon matters more than temperature for enzyme activity in paddy soil. *Soil Biology and Biochemistry*, *135*, 134-143.

Zhang, X., Yang, Y., Zhang, C., Niu, S., Yang, H., Yu, G., ... & Tang, Y. (2018). Contrasting responses of phosphatase kinetic parameters to nitrogen and phosphorus additions in forest soils. *Functional Ecology*, *32*(1), 106-116.
